# Bioactivity of Inhaled Methane and Interactions With Other Biological Gases

**DOI:** 10.3389/fcell.2021.824749

**Published:** 2022-01-07

**Authors:** László Juhász, Szabolcs Péter Tallósy, Anna Nászai, Gabriella Varga, Dániel Érces, Mihály Boros

**Affiliations:** Institute of Surgical Research, Faculty of Medicine, Albert Szent-Györgyi Medical School, University of Szeged, Szeged, Hungary

**Keywords:** normoxia, hypoxia, bioactive gases, methane, mitochondria

## Abstract

A number of studies have demonstrated explicit bioactivity for exogenous methane (CH_4_), even though it is conventionally considered as physiologically inert. Other reports cited in this review have demonstrated that inhaled, normoxic air-CH_4_ mixtures can modulate the in vivo pathways involved in oxidative and nitrosative stress responses and key events of mitochondrial respiration and apoptosis. The overview is divided into two parts, the first being devoted to a brief review of the effects of biologically important gases in the context of hypoxia, while the second part deals with CH_4_ bioactivity. Finally, the consequence of exogenous, normoxic CH_4_ administration is discussed under experimental hypoxia- or ischaemia-linked conditions and in interactions between CH_4_ and other biological gases, with a special emphasis on its versatile effects demonstrated in pulmonary pathologies.

## Introduction

### Respiration From the Atmosphere to the Cells

In the Earth’s atmosphere, where oxygen (O_2_) accounts for ∼ 21% of the environmental gases, reduction-oxidation reactions provide the energy which makes complex organisms capable of sustaining life (Schmidt-Rohr, 2020). Heterotrophs, such as humans, consume organic compounds for energy production by burning O_2_, with carbon dioxide (CO_2_) and water as the ultimate end products. Through this process, the inspired O_2_ level in the lungs is reduced to about 14.5% by the presence of alveolar water vapour and CO_2_, and then the O_2_ levels range from 3.4 to 6.8% by the time it reaches the peripheral tissues (Carreau et al., 2011). Thus, “normoxia” corresponds to the atmospheric O_2_ pressure, and much lower but still physiological (“normal”) levels of O_2_ are found in different tissues within the organs (Carreau et al., 2011). The evolution of aerobic cells has created a range of control mechanisms for the optimal utilization of O_2_ for subcellular, mitochondrial respiration, where multiprotein complexes of the electron transport system (ETS) are dedicated to accepting electrons from reduced carriers and delivering them to accessible molecular O_2_. Three of these complexes (Complex I, III and IV) are also H^+^ channels, responsible for a transmembrane electrochemical gradient between the surfaces of the inner membrane and the resulting driving force for ATP synthase (Complex V), which transforms adenosine diphosphate (ADP) into adenosine triphosphate (ATP).

In this substrate-level oxidative phosphorylation (OxPhos) reaction, availability of O_2_ is the most critical issue. However, many other gases, oxidative or reductive metabolic by-products of this aerobic system can also influence the intra- and extramitochondrial responses. In addition, it is highly likely that the many ways in which the gases combine both in physical and biochemical ways determine the nature of organ responses in clinical conditions associated with hypoxia. The first aim of this review is to summarize the knowledge of certain possibilities through which mitochondrial activity may be modulated by exogenous biological gases, with special emphasis on pulmonary reactions. Indeed, in terms of clinical applications, pulmonary gas delivery is an attractive idea, since applying a bioactive agent either prophylactically or at the time of an operation allows for prompt, specific and local interventions at the barrier sites of the respiratory tract. In this sense, a medically important gas should be easy to apply, have the appropriate chemical and physical properties and kinetics (e.g., be dissolved in plasma), and be nontoxic and biocompatible to achieve the expected biological results. The research on bioactive gases and derivatives has been intense, leading to the listing of four essential characteristics (simplicity, availability, volatility and effectiveness) and the definition of six criteria that make a gas physiologically important or irreplaceable (Wang, 2014). To date, nitrogen monoxide (NO), hydrogen sulphide (H_2_S) and carbon monoxide (CO) are “officially” recognized as signalling substances and referred to as gasotransmitters (Wang, 2014). Against this background, many attempts have concentrated on the therapeutic outcomes of gas deliveries of individual gasotransmitters in various pathological conditions. Nevertheless, the consequences of a more complex interplay of intrapulmonary O_2_ with NO, CO or H_2_S have not yet been investigated systematically.

Further, it should also be taken into account that there are many other gas molecules present in the cellular environment that do not fully meet the gas mediator criteria under the current classifications. Although methane (CH_4_) is conventionally believed to be physiologically inert, studies cited in this review demonstrate that it can modulate the pathways involved in key events of inflammation and influence the interactions of other biological gases. Therefore, the second part deals with CH_4_ bioactivity, a consequence of exogenous, normoxic CH_4_ administration in experimental hypoxic conditions and the implications of its interactions with other gases in respiratory pathologies.

### Subcellular Hypoxia

Hypoxic air induces a number of compensatory responses in the microenvironment of the lung. As the cells become less oxygenated, pulmonary mitochondria have less access to substrates (O_2_ and acetyl-CoA), and the uncontrolled calcium (Ca^2+^) influx is accompanied by reactive oxygen species (ROS) formation (Lukyanova and Kirova, 2015). More importantly, a rapid compensatory mechanism prevents or reverses acute hypoxia-induced disturbances, while a delayed mechanism is responsible for a reversible reprogramming of the regulation of mitochondrial complexes so that the mitochondrial respiratory chain switches from oxidation of NAD-related substrates (Complex I) to succinate oxidation (Complex II), thus providing proper ATP synthesis. Indeed, Complex I contributes to roughly 80% of mitochondrial respiration in normoxia, whereas, during an impeded or deficient O_2_ supply, this is significantly reduced in favour of Complex II [also causing mitochondrial fission through GPR91 signalling (Lu et al., 2018)], which then contributes to nearly 75–90% of the total respiration. Therefore, these hypoxic cells are able to respond in a regulated manner to reduced O_2_ supply; compensatory mechanisms will ensure adequate ATP synthesis until the cellular PO_2_ reaches a critically low ( <1%) level. It follows that a mechanism that allows cells to sense even a minimal change in O_2_ supply activates signalling pathways responsible for triggering adaptive responses. Cytochrome c oxidase (Complex IV) is the main enzyme that transfers electrons and binds O_2_ in the ETS, and thus it was proposed that cells should have O_2_-sensing mechanisms regardless of their bioenergetic state (Bell et al., 2005; Guzy and Schumacker, 2006; Kierans and Taylor, 2021).

**FIGURE 1:**
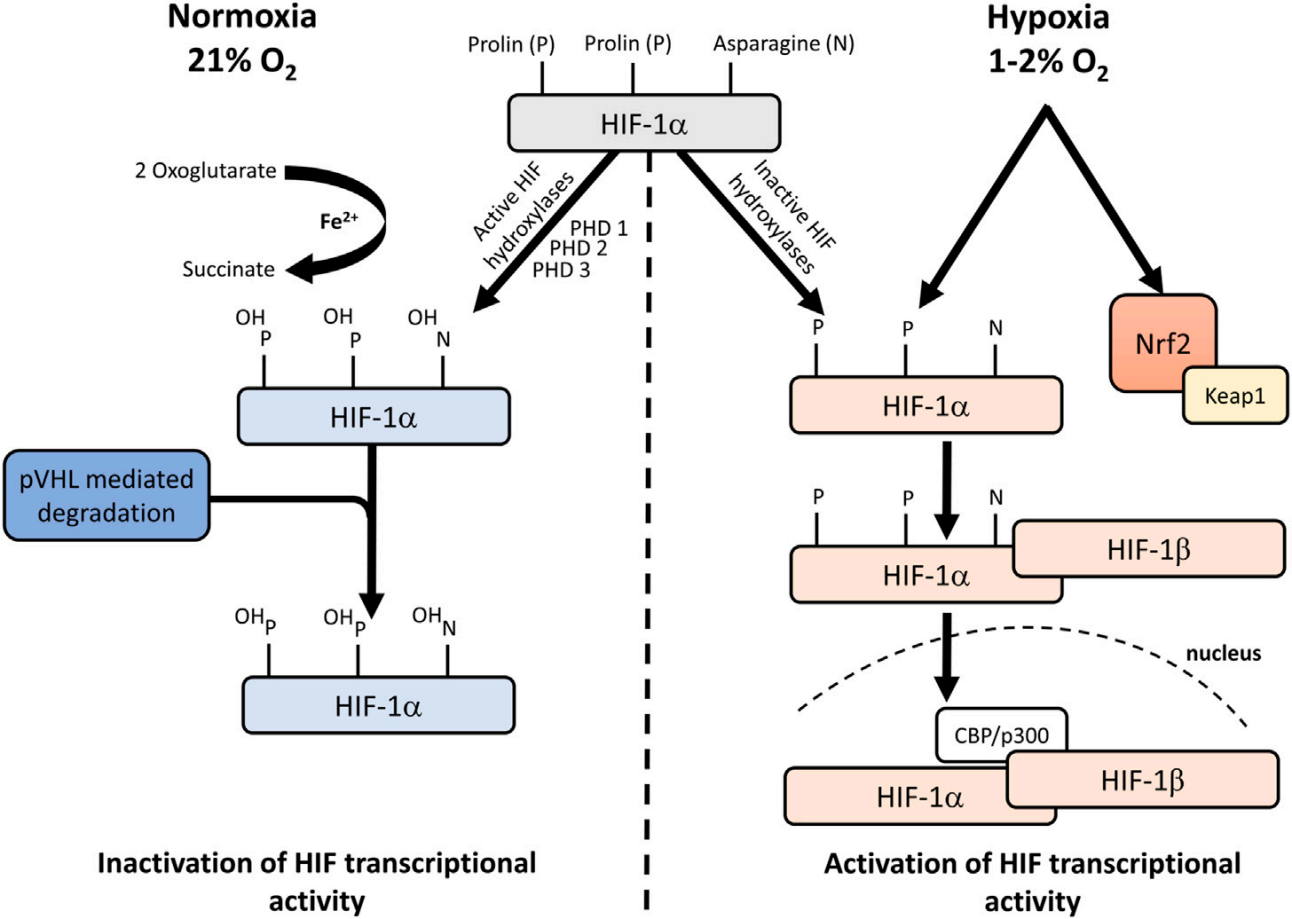
Hypoxic regulation of HIF-1. Under normoxic conditions, NO impairs the activity of HIF-1α prolyl hydroxylases and inhibits HIF-1α ubiquitination and interaction with pVHL. Under hypoxic conditions, NO induces Keap1 signalling and suppresses HIF-1α hypoxic stabilization. CH_4_ increases the expression of Nrf2. HIF: hypoxia-inducible factor; pVHL: von Hippel–Lindau protein; Ub: ubiquitin; NO: nitrogen monoxide; PHD: prolin hydroxylases; Keap1: kelch-like ECH-associated protein-1; Nrf2: nuclear factor-erythroid factor 2-related factor 2.

The transcriptional activator hypoxia-inducible factor (HIF-1) is responsible for regulating oxygenation and is required for the increased expression of more than 60 genes under hypoxia. In aerobic conditions, cells express the COX4-1 regulatory subunit of Complex IV under HIF-1 regulation but switch to the COX4-2 subunit in hypoxic conditions (Semenza, 2011). The stability, subcellular localization and transcriptional activity of HIF-1α are also strongly affected by changes in O_2_ levels. In normoxia, the transcriptional activity of HIF-1α is inhibited by ubiquitous proteases **(** Figure 1 **)**. In this process, HIF-1α can bind to the von Hippel–Lindau tumour suppressor protein (pVHL) after hydroxylation of prolyl (PHD 1,3) (with 2 oxoglurate and Fe^2+^ as cofactors), which promotes ubiquitin-mediated degradation (Bell et al., 2005). In O_2_-deficient states, hydroxylation does not occur, so pVHL cannot bind to HIF-1α, leading to a decrease in degradation processes. In the normoxic state, the binding of the transcription cofactors p300 and CBP to HIF-1α is inhibited so that, in contrast to the hypoxic state, further transcription processes are also prevented (Bell et al., 2005).

HIF-1α is regulated by mitochondria in two different ways. Firstly, if the respiration is inhibited, an intracellular O_2_ level of around 1% is still sufficient for the hydroxylation of HIF-1α. Hydroxylation is only reduced when O_2_ tensions are below 1% so that OxPhos or the ability to respire is not related to the regulation of hypoxic stabilization of HIF-1α. The second is that ROS production during hypoxia is required for HIF-1α protein stabilization (Brunelle et al., 2005). It would logically follow that in hypoxic conditions, ROS formation is reduced in the absence of O_2_, but the levels paradoxically increase during hypoxia (Guzy and Schumacker, 2006). It has been shown that ROS generated at Complex III stabilize HIF-1α during hypoxia (Solaini et al., 2010) and that HIF-1α expression is reduced when Complex V is inhibited with oligomycin (Gong and Agani, 2005). Taken together, the available data do not allow us to clearly establish the exact role of mitochondrial ROS in the regulation of HIF-1α, but the pathway that stabilizes HIF-1α can undoubtedly be considered mitochondria-dependent. According to some authors, mitochondrial ROS can also stabilize HIF-1α under hypoxic conditions (*via* a transcriptional regulatory cascade) *via* the nuclear factor E2-related factor 2 (Nrf2) pathway (Lacher et al., 2018; Potteti et al., 2021).

Together with Complex III and IV, the Complex I (NADH: ubiquinone oxidoreductase) is involved in proton pumping from the matrix to the intermembrane space (extruding four hydrogen ions per NADH). Through this action, Complex I collects the Krebs cycle-derived reducing equivalents and participates in redox energy conversion, and the proton gradient across the membrane is then used for energy production by the ATP synthase during OxPhos (Ramsay, 2019). More importantly, considerable amounts of ROS can be generated by Complex I in the mitochondrial matrix when electrons flow both in the forward (forward electron transport; FET) or reverse (reverse electron transport; RET) direction. To date, flavin mononucleotide (FMN), Q-binding site and the iron–sulphur cluster N2 have been identified in mitochondrial superoxide generation. Although RET was long considered as an *in vitro* phenomenon, the *in vivo* role in ROS generation has recently been demonstrated (Scialò et al., 2017).

Complex I is one of the largest membrane-bound enzymes (1 MDa MW), with a FMN-containing protein and a number of (eight) iron–sulphur centres. The L-shaped structure consists of two major parts, with the components embedded in the inner membrane and the peripheral arm located in the cytoplasm or mitochondrial matrix (Martin and Matyushov, 2017). When a hydride ion is transferred from NADH to FMN at the peripheral arm, two electrons pass through the iron–sulphur clusters (chain of electron transfer cofactors; Fe2S2 and seven Fe4S2; terminal cofactor N2) to ubiquinone (membrane domain), where the proton extrusion is carried out through the membrane. A unique characteristic of Complex I has recently gained much attention as it has been demonstrated that the limited *in vivo* O_2_ availability deactivates Complex I, which is required for the catalytic activity of ETS enzymes (Maklashina et al., 2002; Hernansanz-Agustín et al., 2017). Given a lack of available substrate, Complex I spontaneously forms a deactive (D) form that can be re-activated by exogenous NADH and ubiquinone administration (Galkin and Moncada, 2017; Blaza et al., 2018). The active (A) state catalyses the rapid NADH oxidation at a linear rate, while a lag phase is present during the D→A transition. The lag phase is prolonged at alkaline pH or in the presence of divalent cations, such as Ca^2+^ or Mg^2+^. Most notably, the transition from catalytically active to dormant D form also occurs during acute hypoxia or ischaemia. The biological consequences of the conformational change are not fully mapped, but is has been shown that it fine-tunes ETS, may reduce oxidative/nitrosative stress and switches the NADH:ubiquinone oxidoreductase activity to a sodium-proton (Na^+^/H^+^) antiporter through its hydrophobic membrane-bound domains (ND2, ND4 and ND5 subunits) (Roberts and Hirst, 2012; Babot et al., 2014; Hernansanz-Agustín et al., 2017). In addition, the D-form is more sensitive to ischaemia/reperfusion (IR)-mediated oxidative injury than the A-form. Therefore, modulation of the dormant form may also be a protective strategy during ischaemia/hypoxia (Chouchani et al., 2013; Gorenkova et al., 2013).

### Hypoxia and Inhaled Bioactive Gases

Although inhaled NO has been successfully tested in neonates and adult patients with acute respiratory distress syndrome (Feng et al., 2021; Lotz et al., 2021; Safaee Fakhr et al., 2021), the clinical benefit of intrapulmonary administration is still subject to much debate (Sokol et al., 2016; Vieira et al., 2021). In this line, the oxygenation of the tissues is a main factor when the rather controversial results of gasotransmitter reactions are discussed. For example, the most important physiological mechanism linked to NO metabolism requires proper O_2_ concentrations; under normal or higher O_2_ tension, the half-life of NO is shorter, while in hypoxic environments NO will be eliminated after a significantly longer time with a number of prolonged effects (Kuschman et al., 2021). Likewise, a combination of low O_2_ tension with mitochondria-derived ROS and higher NO flows leads to peroxynitrite formation (Thomas et al., 2008) with nitroxidative stress and post-translational protein modification (Campolo et al., 2020). Further, like other inhibitors of mitochondrial respiration, NO prevents the stabilization of HIF-1α. A recent key finding has revealed a novel role for Complex I in this process, as prior A/D conversion is necessary for S-nitrosothiols and peroxynitrite to interfere with the respiratory activity of mitochondria (Babot et al., 2014; Galkin and Moncada, 2017). Therefore, the hypoxia-linked mitochondrial duality may explain, at least partly, the controversial clinical results and the narrow range of effectiveness of NO inhalation.

H_2_S is the next gas mediator, with Janus-faced characteristics being clearly present at the mitochondrial level. It is toxic when inhaled in high concentrations, while it is anti-inflammatory and cytoprotective at low partial pressure (Elrod et al., 2007; Cui et al., 2016; Scheid et al., 2021). Inhalation of 80–150 ppm H_2_S induces a suspended animation state with reduced metabolic rate, which leads to an increased resistance to severe hypoxia (5% FiO_2_) (Blackstone and Roth, 2007). In an oxygenated environment and in low (less than 1 µM) H_2_S concentrations, the regular substrates of the respiratory chain are used for biological oxidation. However, as soon as H_2_S content is increased (to less than 10 µM) an active sulphide quinone reductase (as the immediate electron acceptor) is available, H_2_S acts as an alternative electron source for the respiratory chain. A H_2_S concentration of over 10 µM impairs the mitochondrial function with the inhibition of Complex IV (Bouillaud and Blachier, 2011). Here it should be added that cancer cells may utilize this phenomenon by up-regulating H_2_S, thus producing enzymes to stimulate mitochondrial ATP synthesis and maintain mitochondrial function (Szabo, 2021).

Carbon monoxide (CO) is the third gas in the sequence of gasotransmitters, again with dual properties: low levels exert cyto- and tissue protective effects, but in higher concentrations systemic toxicity comes to the fore. Due to its affinity to bind to the haem iron centre of haemoglobin, carboxyhaemoglobin (CO-Hb) formation ensues with cellular hypoxia. A number of signal transduction pathways have been recognized as potential targets of low concentrations of inhaled CO *via* its anti-inflammatory (Otterbein et al., 2000), anti-apoptotic (Ryter et al., 2018), anti-oxidative (Parfenova et al., 2012) and anti-proliferative effects. CO binds primarily to haem iron and may activate soluble guanylate cyclase, although with lower efficacy than NO (Sharma and Magde, 1999). Through the modulation of the mitogen-activated protein kinase pathway, CO inhibits the expression of several pro-inflammatory cytokines, such as tumour necrosis factor-alpha (TNF-⍺) and interleukin-1beta (IL-1β), and increases the expression of the anti-inflammatory cytokine interleukin-10 (IL-10) (Otterbein et al., 2000). The mitochondrium is also one of the recognized cellular targets for CO, with physiological concentrations of CO increasing mitochondrial ROS generation, which activates cellular endogenous mechanisms of defence involved in preconditioning and cytoprotection (Bilban et al., 2008). Furthermore, CO prevents apoptotic cell death by limiting mitochondrial membrane permeabilization, which inhibits the release of pro-apoptotic factors into the cytosol; both events are ROS-dependent (R. Oliveira et al., 2016). The protective effects of low concentrations (of up to 500 ppm) of inhaled CO have been observed in a number of lung injury models (Otterbein et al., 1999; Morse et al., 2003; Kohmoto et al., 2006). However, most of the clinical studies in various disease trials have been terminated because the expected primary outcomes had not been met.

### Inhaled CH_4_

**FIGURE 2:**
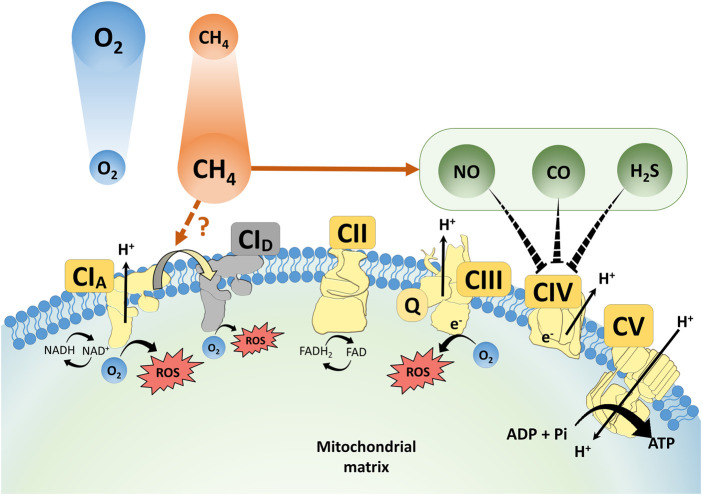
A scheme for the proposed interaction of CH_4_, NO, CO and H_2_S at mitochondrial respiratory complexes. ROS: reactive oxygen species; NO: nitrogen monoxide; CO: carbon monoxide; NAD/NADH: nicotinamide adenine dinucleotide/dihydronicotinamide adenine dinucleotide; CI–V: Complex I–V; TCA cycle: tricarboxylic acid cycle; FAD/FADH_2_: flavin adenine dinucleotide/dihydroflavine-adenine dinucleotide; Q: ubiquinone.

The bioactivity of all recognized gas mediators is related to their tendency to react chemically with biologically important target molecules. Therefore, and precisely due to this characteristic, these compounds are also categorized as toxic asphyxiants in environmental chemistry. For example, and according to current knowledge on NO, CO and H_2_S biochemistry, these gaseous substances all readily inhibit mitochondrial O_2_ consumption by Complex IV (Figure 2). It is therefore important to consider that physiologically important gases that trigger vital functional changes will have profound adverse effects in any cellular system given sufficient exposure, and many of the unfavourable consequences are directly linked to inhibition of mitochondrial function.

On the other hand, simple asphyxiants, such as CH_4_, act by physically limiting the utilization of O_2_, without producing cytotoxic effects (Boros et al., 2018). Tissue hypoxia may indeed occur when CH_4_ displaces the air and hence O_2_ in a restricted space. However, in such cases, respiratory distress is not due to the chemical specificity of the gas, but to the decreased O_2_ content (CH_4_ in the inspired air should be present at about 14% or 140,000 parts per million by volume (ppmv) to reduce O_2_ to 18%) (Boros and Keppler, 2019). Information on the respiratory effects of CH_4_ is sparse but the inhalation of normoxic artificial air containing 21% O_2_ and 2.5% CH_4_ had no side-effects on the blood gas chemistry and mean arterial blood pressure in normotensive unstressed animals (Boros et al., 2012; Zaorska et al., 2021). Likewise, the administration of CH_4_-enriched saline did not affect cytochrome c release in rats (Wang et al., 2017).

Under standard conditions for temperature and pressure, the solubility of CH_4_ in blood is rather low (a blood:air partition coefficient of 0.066) but significantly higher in membrane bilayers (a partition coefficient of 0.20) [as reviewed by Boros and Keppler (2018)]. Therefore, the concentration of CH_4_ in the tissues rapidly reaches equilibrium with that in the inspired air, and this equilibrium remains stable even with prolonged exposure time (Watanabe and Morita, 1998). It follows that inhaled CH_4_ will move readily from the alveoli into the circulation, throughout which it is distributed rapidly and may accumulate transiently at cell membrane interfaces, thereby changing the relationship between gases and the *in situ* functionality within this environment. Without a new exogenous supply, CH_4_ will enter the circulation again and then be excreted through the lungs if its partial pressure is higher than that in the atmosphere.

The outcome of exogenous CH_4_ respiration in the human body under stress conditions has not yet been evaluated. Nevertheless, a wealth of data is available in plants and animals in such situations and also on the links between CH_4_ and gas messengers. The effects of CH_4_ supplementation to CO, H_2_S and NO biology were repeatedly shown during the adaptation to abiotic stress and germination inhibition in plant species, which confirmed that CO, NO and H_2_S signalling mechanisms are involved in the molecular basis of CH_4_-induced stress tolerance (Cui et al., 2015; Qi et al., 2017; Kou et al., 2018). Apart from plant pathophysiology, several series of *in vivo* analyses have demonstrated that CH_4_-containing normoxic artificial air has anti-inflammatory effects by decreasing the biochemical, functional and structural consequences of nitroxidative stress [(Boros et al., 2012)**,** (Mészáros et al., 2017a)**,** (Poles et al., 2018)**]**. Data show that NO can directly inhibit mitochondrial functions *via* several pathways and that NO-influenced or mediated inhibition can be reversed with 2.2–2.5 %v/v CH_4_-containing gas mixtures. Notably, it has been demonstrated that normoxic CH_4_ ventilation decreases tyrosine nitrosylation after IR injury, a process which involved NO and peroxynitrite formation. In addition, exogenous CH_4_ administration reduced the xanthine oxidoreductase (XOR)-linked nitrate reductase activity, the generation of nitrogen-centred radicals and the damage to nitrergic neurons during a standardized IR challenge (Poles et al., 2018). Along these lines, it has been shown that higher concentrations of exogenous CH_4_ can lead to direct anti-cytokine effects *via* master switches, such as Nrf2/Keap1 and NF-κB (Mészáros et al., 2017b). More recently, the addition of 2.5% v/v CH_4_-normoxic air mixture to the oxygenator sweep gas reduced the systemic inflammatory response to extracorporeal circulation in a clinically relevant large animal model. In this study, the inotropic demand was significantly lower, the renal arterial flow was significantly higher, and the hour diuresis remained in the low normal range as compared to the oliguria in the non-treated animals (Bari et al., 2019) (Supplementary Table S1).

In this line, many studies have also explored the relationship among CH_4_ actions in the context of mitochondrial biology. Inhaled CH_4_ reduced cytochrome c release and preserved the mitochondrial respiratory capacity *in vivo* and in transient anoxia-treated cell cultures as well (Strifler et al., 2016; Jász et al., 2021). Recently, we carried out a sequential study with exogenous normoxic CH_4_ in simulated IR environments using a high-resolution respirometry system to quantify the ETS responses (Jász et al., 2021). In this protocol, CH_4_ treatment restricted the forward electron transfer within Complex I in control mitochondria while effectively restricting RET in post-anoxic mitochondria, thus it could be concluded that interaction with Complex I occupies a key position in the protective mechanism of CH_4_ against a hypoxia/reoxygenation injury (Jász et al., 2021). Parallel *in vivo* studies have also shown that the CH_4_ content of an organ preservation solution effectively influenced several components of the endoplasmic reticulum (ER) stress-mitochondria-related pro-apoptotic signalling pathways (Benke et al., 2021). The myocardial OxPhos capacity was more preserved and cytochrome c release was decreased as a result of CH_4_-enriched storage, with the relative mRNA expression for hypoxia- and ER stress-associated genes (including HIF-1α) also being significantly reduced (Benke et al., 2021). Indeed, several previous studies demonstrated that exogenous CH_4_ modulates the intrinsic, mitochondrial pathway of pro-apoptotic activation in model experiments (Ye et al., 2015; Chen et al., 2016; Liu et al., 2016; Jia et al., 2018) and CH_4_ administration exhibited anti-apoptotic effects and protected the pulmonary epithelial cells in a murine model of ovalbumin-induced allergic asthma as well (Zhang et al., 2019). More importantly, the anti-apoptotic properties of CH_4_ inhalation were associated with improved pulmonary compliance and surfactant production in a rodent model of lung IR injury (Zhang et al., 2021). In summary, a possible indirect way in which CH_4_ supplementation modulates apoptosis is by reducing cytochrome c release from the inner mitochondrial membrane, which has already been demonstrated in several tissues (Chen et al., 2016; Strifler et al., 2016; Wang et al., 2017). It seems that further knowledge of inhaled CH_4_ and other gaseous molecular species with their mitochondrial targets, most importantly of Complex I, has the potential to increase the understanding of the mechanism of pathological processes at work in the pulmonary alveoli and capillaries (Figure 2).

## Discussion and Conclusion

Beyond O_2_ and CO_2_, many gases are biologically active. Signalling roles were demonstrated for NO, CO and H_2_S and it has become clear that these simple, volatile molecules can influence the cellular biology in various ways. Likewise, the human diagnostic relevance of detection of exhaled gases, as signatures of oxido-reductive stress responses, is emerging as well (Paardekooper et al., 2017). Several aspects of mitochondrial respiration, such as energy production, Ca^2+^ homeostasis and intrinsic apoptosis, may also be targets of intertwined gaseous pathways but it is less clear how mitochondrial functions are altered if the membership of this molecular club changes and how the signals of a mixed gaseous input are translated to downstream manifestations of cellular reactions.

As the examples illustrate, bioactivity is not limited to those gases that have inherited the textbook characteristics of gasotransmitters. There is ample evidence that other, less prominent components of the endogenous gaseous network, such as molecular hydrogen (H_2_) or CH_4_, are also able to modulate mitochondrial respiration [(Mészáros et al., 2017b), (Hirano et al., 2021)]. As an analogy, other gaseous compounds, such as NO, H_2_S and CO, were previously thought to be toxic pollutants without any physiologic effects in eukaryotes. CH_4_ has a long evolutionary history on Earth (Hancock, 2017). It is permanent part of the gaseous environment, a nontoxic asphyxiant, which can change the symbiosis with other gas molecules within the internal milieu of aerobic cells. In this scheme, the recognized bioactivity suggests a role for exogenous CH_4_ to modulate the hypoxia-linked pro-inflammatory signals towards resting conditions.
