# Supplementary material for: Bioactivity of Inhaled Methane and Interactions With Other Biological Gases
Source: Front Cell Dev Biol. 2022 Jan 7;9:824749. doi: 10.3389/fcell.2021.824749 (PMC8777024; doi:10.3389/fcell.2021.824749)
Supplement: Supplementary file 1 [file Table1.docx]

**Supplementary Table 1.** **A summary of systemic and local pulmonary effects of inhaled CH_4_**

| **Effect/mechanism** | **Experimental model** | **Dose/concentration** | **Mode of administration** | **Reference** |
| --- | --- | --- | --- | --- |
| Anti-inflammatory properties; decreased ROS/ RNS production, inhibition of leukocyte activation | Canine intestinal ischemia-reperfusion (IR) | 2.5% in air | Inhalation of normoxic air-CH_4_ mixture | Boros et *al.* (2012)  *Crit Care Med* |
| Preservation of the epithelial barrier function; decreased ROS/RNS and improved microcirculation | Rat intestinal IR | 2.2% in air | Inhalation of normoxic CH_4_-air mixture (during 15 min ischemia, reperfusion 60 min) | Mészáros et *al.* (2017)  *Surgery* |
| Small intestinal neuroprotection; inhibition of xanthine oxidoreductase (XOR)-linked nitrate reductase activity, decreased RNS | Rat intestinal IR | 2.2% in air | Inhalation of normoxic CH_4_-air mixture, ischemia 10 min, reperfusion 5 min | Poles et *al.* (2018)  *Free Radic Biol Med* |
| Reduction in systemic inflammatory response; decreased ROS, XOR activity | Systemic inflammatory response, pig model of extracorporeal circulation | 2.5% in air | CH_4_-normoxic air mixture (1 l/min) added to the oxygenator sweep gas) | Bari et *al.* (2019)  *J Cardiothorac Surg* |
| Attenuation of hepatic mitochondrial ETS dysfunction; reduced oxidative damage | Rat liver IR | 2.2% in air | Inhalation of normoxic CH_4_-air mixture, 10 min during ischemia and 60 min reperfusion | Strifler et *al.* (2016)  *PLoS One* |
| Reduction of anoxia-reoxygenation-induced mitochondrial dysfunction and cardiomyocyte injury | In vitro heart IR (cultured cardiomyocytes) | 2.2% in air | CH_4_-artificial air mixture, 2h during reoxygenation | Jász et *al.* (2021)  *J Cell Mol Med* |
| Improvement of graft function; improved mitochondrial function, reduced ER-stress and inflammatory activation | Rat heart transplantation | 6.57±0.27 µmol/ml | CH_4_-enriched cold preservation solution | Benke et *al.* (2021)  *J Heart Lung Transplant* |
| Decreased inflammatory response and apoptosis, improved lung function through Nrf2-mediated pulmonary surfactant regulation | Rat lung IR | 2.5% CH_4_ + 40% O_2_ + 57.5% N_2_ | Inhalation 10 min before the end of the 90-min ischemic period until 60 min reperfusion | Zhang et *al.* (2021) *Front Physiol* |
| Neuroprotection via antioxidative and anti-inflammatory pathways; reduction of MDA and TNF-α levels | Rat middle cerebral artery occlusion-reperfusion | 2.2% in air | Inhalation started during 2 h ischemia and continued during reperfusion | Zhang et *al.* (2017) *Arch Med Res* |
